# Supplementary material for: Modeling spatial contrast sensitivity in responses of primate retinal ganglion cells to natural movies
Source: PLoS Comput Biol. 2026 Apr 7;22(4):e1014157. doi: 10.1371/journal.pcbi.1014157 (PMC13099094; doi:10.1371/journal.pcbi.1014157)
Supplement: Attachment — Submitted filename: point_by_point_response.pdf [file pcbi.1014157.s001.pdf]

We thank the two Reviewers and the Editors for handling our manuscript and for the constructive comments. We have addressed all points raised by the Reviewers as specified below (in blue). The changes to the manuscript can also be inspected in the tracked-manuscript version that we supply. The line numbers indicated below refer to the lines in the clean manuscript version without track changes.

Reviewer #1:

This manuscript presents a new approach for characterizing neural feature selectivity by incorporating selectivity to contrast. The method aims to find a compromise between the more complex subunit models and the models based on single stimulus component. The authors show that adding selectivity to contrast improves encoding accuracy under while noise stimulation but not natural stimulation. In the same of natural stimuli, authors note that because they are dominated by low spatial frequencies, there is relative lack of contrast at small scales, leading to the absence of improvement.

We thank the Reviewer for the careful evaluation of our work. Please note, however, that for natural stimuli there is not an absence of improvement by including spatial contrast information (at least for analyzed cell types other than the OFF midget cells), but merely a reduction in the improvement. Where we did observe a lack of improvement for natural stimuli was the smoothing analysis of Fig. 7 (Fig. 6 in the previous version), that is, evaluating the local spatial contrast after stimulus smoothing to cut off contrast at very fine spatial scales. Here, however, the baseline is the spatial contrast model with no smoothing, not the LN model with no spatial contrast information. In the revised manuscript, we have clarified these aspects (lines 395–400 and lines 643–649) to avoid this misunderstanding.

To substantiate the main claim that this method offers an improvement relative to subunit models it is necessary to include comparison with at least one of the subunit-based methods for characterizing neural feature selectivity.

As suggested, we now compare the spatial contrast (SC) model to a subunit model that we fit to the data. To obtain subunits, we perform spike-triggered covariance analysis on the data from the white-noise recordings (as suggested by the Reviewer in the comment below), after integrating out the temporal dimension. To turn the extracted filters into subunits, we follow the procedure suggested by Kaardal et al (Neural Comput 2013; 25:1870–1890), which yields well-defined, spatially localized subunits for our data. The subunits form the basis of a parameterized subunit model with parameter optimization performed on the training data, as for the SC model. The subunit analysis and the performance comparison with the SC model is included in a new figure (Fig. 6), which shows that the SC model performs not worse but rather better than the subunit model. Although further optimization of the subunit model might alleviate this problem, the analysis shows that the SC model is not easily outperformed by a subunit model and underscores that the simplicity of the model serves the purpose of an easy-to-obtain, yet reliable response prediction with nonlinear spatial integration. The subunit-model analysis including the interpretation of its performance is presented in a new section of the manuscript (lines 299-347).

Another substantial concern is that perhaps contrast needs to be evaluated not over the receptive field but at a small scales (i.e. subunits) and then combined according to an optimized combination. This would provide a more direct link to subunit models. This also can impact comparison in the case of the natural scenes, because contrast can be evaluated over small scales.

Thanks for this comment, which allows us to explain the influence of spatial contrast at different spatial scales and the similarity of the SC model to subunit models in this respect in more detail. In the original form (that is, without spatial stimulus smoothing as considered in Fig. 7 in the revised manuscript version), the SC model is sensitive to spatial contrast on any scale below the size of the receptive field (and above the size of stimulus pixels), as any variation of pixel intensities within the receptive field contributes to the local spatial contrast measure. Thus the model does not evaluate spatial contrast at one particular scale (of the receptive field), but at all finer scales, the same as for a subunit model with subunits corresponding to individual stimulus pixels. Clearly, however, subunits are expected to be larger than individual stimulus pixels, and this is where the spatial smoothing of Fig. 7 comes into play. The smoothing cuts off spatial contrast at high spatial frequency, and for a subunit model, the optimal cutoff should be given by the size of the subunits. Thus, the SC model is sensitive

to spatial contrast at the scales of subunits, and the smoothing analysis allows a direct link to subunit models (e.g., expecting the derived optimal spatial scale to reflect the subunit size), as suggested by the Reviewer. In the revision, we have clarified these aspects, in particular the link to subunit models, at several places (e.g., lines 52–54, 356–358, 363–366, and 581–588) and also laid out the connection to spatial contrast in natural stimuli more clearly (lines 640–649).

Please note that some subunit models are not technically demanding and can be performed based on spike-triggered covariance.

Indeed, we have followed the Reviewer's suggestion and based the subunit-model analysis on spike-triggered covariance analysis, as described in our response to the point above.

The paragraph on lines 254-260 about off midget cell type benefiting least from the inclusion of contrast information contradicts the data: technically off parasols benefit the least.

We have revised the statement to now state that OFF midget cells profit little (not necessarily least) from spatial contrast information (lines 253–257). The comparison with other cell types is not relevant at this point. Note, though, that for natural movies, OFF midget cells clearly have the smallest relative performance increase on average by including spatial contrast information. For white noise, the situation is indeed more complicated. Overall, the relative performance increases are comparable, but for the dataset where both cell types have been observed, the relative performance increase is considerably larger for OFF parasol cells.

Discussion regarding comparison with natural scenes (Sec. 3.5) is problematic. It implies that subunits are not relevant under natural conditions. But a more likely explanation lies with the suboptimality of the scale over which contrast was estimated, and perhaps also in the temporal domain.

We apologize for our apparently unclear wording. The lack of high-frequency spatial contrast in natural scenes does not mean that spatial contrast information or subunits would not be relevant, but that the relevant spatial contrast in the stimulus is at relatively "low" spatial frequencies, that is, at scales larger than the subunits, but still smaller than the receptive field. Thus, smoothing out spatial scales below the subunit level has no effect, in contrast to the case of white-noise stimulation. We have clarified this now in the Discussion (lines 643–649) as well as in the relevant section of Results (lines 395–400).

Reviewer #2:

The article by Sridhar et al. studied how retinal ganglion cell responses to natural movies of the marmoset ex-vivo retina could be modeled with a spatial contrast (SC) term in a model with few parameters (4). This represents an extension of the work presented by some of the authors in Liu et al. 2022, where they introduced the model to predict ganglion cell responses to static natural images. The study is well written and presented. The figures are clear. As the authors state, this is an incremental work from what has been published, but it was an important step to prove it. They also acknowledge that these are not state-of-the-art models to predict ganglion cell responses, but that they represent a good benchmark, better than the classical LN, which is as easy to fit, yet explains more of the cell activity.

We thank the Reviewer for the careful evaluation of our work and for the supportive comments.

My main remark is that the authors did not explain the reason for switching to marmoset retina, instead of testing their model in salamander and mouse retina as in the previous article. Second, although it is clear that SC is used by the cells more than just mean intensity, the claim that a 4- vs. a 3-parameter model has better performance should be better supported. Did they try any other second-order statistic besides SC (homogeneity, angular second moment, correlation, entropy?) to show it is actually the one encoded by the cells?

Thanks for this comment, which allows us to clarify the motivation of our approach further. Using the primate retina in the present article was, first of all, made possible by a recent opportunity to record from marmoset retinas and primarily motivated by the goal to demonstrate the usefulness of the concept of including spatial contrast information for modeling also in the primate retina. Together with

the extension of the modeling to spatiotemporal and natural movies, we believe that this adds to the novelty of the manuscript, in particular since it allows us to relate our findings to known and identifiable subtypes of ganglion cells, which would not have been readily possible, for example, in the salamander retina. We have changed the wording in the Introduction (lines 46–48) and when discussing cell-type effects in the Discussion (lines 589–591) to better reflect this.

Regarding alternative second-order statistics, we have now added a paragraph in Discussion (lines 453–460), which explains our choice of the LSC. We considered other possibilities but these either would be highly correlated with the used LSC (but less straightforward) or difficult to make independent of the mean-intensity signal.

Remarks:

1. Figure 2 is technically informative, but it lacks real biological interest. As it is, this figure is not much more informative than the equations. Also us and hs are not defined in the caption.
If the claim is that this cell is responding to high contrast:
◦ Why not superimpose the contrast and the Imean traces calculated on top of the firing rate responses to exemplify that (accounting for latency) the cell responds to high values of one of them?
◦ Why not use a local peak of the contrast or intensity curve to exemplify the stimulus (and convolved stimulus) that the cell is responding to? In fact, this question comes from not knowing if the value of 0.33 is big (or huge) for making the cell respond (or maybe there is some coupling needed between Imean and SC?). Maybe give some reference values? It is very unclear why the values selected are relevant to understand better, or whether they are just random examples.
◦ Then it would be very nice to see two cell examples behaving differently: I would add the OFF midget example here.
◦ Related, line 197: You really need the evolution over time of LSC superimposed on the FR curve to clearly see this relation; and line 203: This is too simplified, we need to see the evolution of the Imean and LSC curves together with FR.
◦ I would also add these curves to Figure 5, maybe not superimposed on the existing curves, but above or below.

Thanks for the suggestions. We agree and have largely followed the Reviewer's suggestions for revising the figures and adding information about $\mathrm{I}_{\mathrm{mean}}$ and LSC. In Fig. 2, we now include traces of the two variables to better exemplify the difference in LSC for the two sample cases. We give their values in the figures primarily as an illustration that, for a given point in time, each measure contributes a single value and that these values are very similar for $\mathrm{I}_{\mathrm{mean}}$ for the two samples, but differ for LSC. In the traces, scales are now given by z-scoring the values so that units correspond to standard deviations of the range of $\mathrm{I}_{\mathrm{mean}}$ and LSC. As suggested, we now also added a second example, which comes from an OFF midget cell and specify u_s and h_s in the caption. Finally, in Fig. 5, we also added the traces of $\mathrm{I}_{\mathrm{mean}}$ and LSC to underscore how local spatial contrast contributes to the cell's activation.

2. Line 191: Did you quantify RF size or any other parameter across datasets to discard/find which could be involved in this discrepancy?

Yes, we compared receptive field sizes across the two recording sessions but did not find substantial differences (e.g., 118 µm ± 12 µm versus 115 µm ± 9 µm, mean ± standard deviation, for OFF parasol cells, the only specific type consistently found in both datasets) nor any other obvious deviations across the two datasets. We have added this information to the manuscript (lines 170–173). Note, though, that variations of receptive field sizes with eccentricity become fairly small in the far periphery (e.g., Masri et al, J Comp Neurol 2019; 527:236–258). We therefore can only speculate that eccentricity or experimental variability is behind the differences in the datasets, as indicated in the text.

3. Line 228: Is the decision to only weight the model with w on LSC based on previous knowledge that there are not pure LSC-coding cells? Or is it possible to have a huge w that disregards Imean? An option to not increase the number of parameters would be to have w1 and w2 with a constraint for them to sum to 1.

We had considered using weights w and $1 - w$ for $\mathrm{I}_{\mathrm{mean}}$ and LSC, but decided to go with the current

form simply to be conceptually closer to an LN model with added spatial contrast sensitivity. We don't expect and don't observe cells that would be purely LSC encoders, which would be detectable by a very large weight w. In such a case, one could switch to the otherwise equivalent weight parameterization mentioned above. We now explain this when introducing the model details in Methods (lines 1004–1009).

4. Line 234: Would the model be even better if you allow some variation of this Gaussian fit? Also, to avoid over-parameterization one may use sigmax and sigmay plus an ellipse angle. I wonder if Imean and LSC may be integrated over different spatial scales. A comment about this is present in line 405, but the justification to argue that they are probably equal is not well explained.

This is an interesting idea, which, in fact, we are currently pursuing for follow-up work. Note, though, that fitting the filter as part of the model optimization substantially complicates the procedure, as the filter outputs I_{mean} and LSC then need to be recomputed for the entire (long) stimulus set at every iteration of the optimization algorithm. Here, however, our goal is to provide a model that can be readily used with little computational demand. Thus, in the presented form, the model can even be used if the dataset to be modeled is too small to estimate a receptive field (say, a short movie or a limited set of images) or if information about the receptive field is obtained by other means, e.g., through flashed spots as often done in patch-clamp experiments. Moreover, our results so far suggest that fitting the receptive field alongside with the other parameters of the SC model does not provide a measurable benefit, at least for white-noise stimuli, as is expected because the spike-triggered average is a good filter for the LN model and the LN model is the I_{mean} part of the SC model. Let us further note that allowing for different filters for I_{mean} and LSC complicates the model in an interesting and fundamental way because the computation of the pixel standard deviation includes the subtraction of the mean, which couples the computation of I_{mean} and LSC. In the revised manuscript, we now better explain these points when presenting SC model results (lines 225–229) and when arguing for the plausibility of using a single filter in the Discussion (lines 491–507).

5. Line 324: What do you mean that the models would benefit by properly assessing SC? Was it poorly assessed? Are there better options?

We apologize that our wording was apparently unclear. With "properly", we meant using the right range of spatial scales, that is, the right level of spatial smoothing. We have now specified this in the revised test (lines 389–391).

6. One main claim is that they can estimate the spatial scale at which the cells integrate information from the movies. It may be possible to compare this with the scales encountered with static images (taking into account the different species)?

Thanks for this comment, which prompted us to revisit how we introduce and explain the spatial scale of nonlinear stimulus integration. We now note that this is not a single scale of integration, but should be considered more a threshold above which spatial contrast is important because it is not averaged out (lines 363–366 and 581–588). In this context, our repeated use of "optimal scale" may have been misleading, as this only refers to optimization of response prediction by the model, not an optimal scale in terms of information processing or behavioral tasks. We therefore revised the terminology throughout the text. We now also discuss the relation to scales encountered in natural scenes, but given the scale invariance of natural images and the dependence of integration scales in the outside world, we argue that the observed scales of nonlinear stimulus integration more likely reflect biological constraints than adaptation to scene statistics (lines 606–612).

Minor comments:

- *It is confusing to cite Fig. 1B before Fig. 1A.*

We have rearranged the text and now cite Fig. 1A before 1B.

- *Line 123: It is not clear if this is known or if it is shown for the first time here. Maybe a citation is needed?*

We apologize for the lack of clarity here. The different degrees of nonlinearity have been reported previously, and we have now included relevant citations (lines 80–82).

- *Line 124: Change "displayed" to "display" (as I understand this is a general remark; if not, rephrase to make it clear it refers to your data).*

Done. (Line 83.)

- *Red and black superimposed traces are not colorblind friendly (Fig. 5 and Fig. 6C).*

Thanks. We now use a different color scheme and checked that this is colorblind friendly.

- *Line 135: A citation or a reference to the Methods is needed to justify this stimulus.*

We have slightly expanded this to justify the construction of the stimulus and now also reference the Methods and a citation (lines 94–98).

- *Lines 495/496: I think there may be a typo in the second "marmoset"; I think it refers to macaque.*

Indeed! Thanks a lot for picking this up. Corrected now (line 631).
